# Effects of age, sex, and genotype on high-sensitivity metabolomic profiles in the fruit fly, *Drosophila melanogaster*

**DOI:** 10.1111/acel.12215

**Published:** 2014-03-18

**Authors:** Jessica M Hoffman, Quinlyn A Soltow, Shuzhao Li, Alfire Sidik, Dean P Jones, Daniel E L Promislow

**Affiliations:** 1 Department of Genetics, University of GeorgiaAthens, GA, 30602, USA; 2 Division of Pulmonary Allergy & Critical Care Medicine, Department of Medicine, Emory UniversityAtlanta, GA, 30322, USA; 3 Department of Medicine, Clinical Biomarkers Laboratory, Emory UniversityAtlanta, GA, 30322, USA; 4 ClinMet Inc.3210 Merryfield Row, San Diego, CA, 92121, USA; 5 Center for Health Discovery & Well Being, Emory UniversityAtlanta, GA, 30322, USA; 6 Department of Molecular Biosciences, University of TexasAustin, TX, 78712, USA; 7 Department of Pathology and Department of Biology, University of WashingtonSeattle, WA, 98195, USA

**Keywords:** age, aging, *Drosophila melanogaster*, genetic variation, genotype, heritability, metabolomics, sex, systems biology

## Abstract

Researchers have used whole-genome sequencing and gene expression profiling to identify genes associated with age, in the hope of understanding the underlying mechanisms of senescence. But there is a substantial gap from variation in gene sequences and expression levels to variation in age or life expectancy. In an attempt to bridge this gap, here we describe the effects of age, sex, genotype, and their interactions on high-sensitivity metabolomic profiles in the fruit fly, *Drosophila melanogaster*. Among the 6800 features analyzed, we found that over one-quarter of all metabolites were significantly associated with age, sex, genotype, or their interactions, and multivariate analysis shows that individual metabolomic profiles are highly predictive of these traits. Using a metabolomic equivalent of gene set enrichment analysis, we identified numerous metabolic pathways that were enriched among metabolites associated with age, sex, and genotype, including pathways involving sugar and glycerophospholipid metabolism, neurotransmitters, amino acids, and the carnitine shuttle. Our results suggest that high-sensitivity metabolomic studies have excellent potential not only to reveal mechanisms that lead to senescence, but also to help us understand differences in patterns of aging among genotypes and between males and females.

## Introduction

Lifespan is a highly heritable trait. Over the past 20 years, researchers working on lab-adapted organisms have been able to identify evolutionarily conserved genetic pathways which, when knocked down or overexpressed, are able to dramatically increase lifespan. These successes underscore two critical questions: first, at the molecular level, what are the underlying mechanisms by which these genes affect longevity; second, at the population level, do these same genes account for standing variation in longevity in natural populations?

These questions are complicated by the fact that the age at which an individual dies depends not only on its genotype, but also on a lifetime of effects accumulated through environmental exposure, the environment-specific response of genes, and the downstream physiological consequences of these complex factors. Fortunately, whole-genome sequencing and genome-wide association (GWA) studies now make it possible to identify segregating alleles that affect complex phenotypes such as body height, diabetes, schizophrenia, and even longevity (Jeck *et al*., 2012), but GWA studies suffer from numerous challenges, and these are further compounded in analyses of lifespan. First, alleles identified in GWA studies typically explain just 0.1–1.0% of the variation in complex traits (Park *et al*., 2010). Second, the genetic basis of lifespan appears, at least in part, to differ between the sexes (Burger & Promislow, 2004). Third, lifespan includes a substantial degree of stochasticity, varying dramatically even among genetically identical individuals raised in a constant and identical environment (Kirkwood *et al*., 2005). Finally, and perhaps most importantly, lifespan is a highly composite trait potentially influenced by the functional decline of many underlying processes. To fully understand the genetics of lifespan, we need to understand the genetics not simply of age at death, but rather of the underlying causes of death.

**Figure 1:**
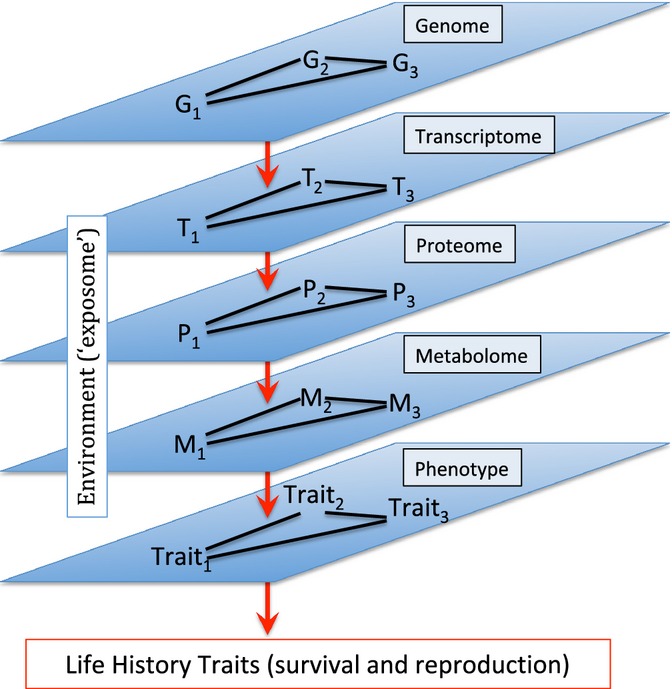
To complete the map from genotype–phenotype (G-P), we face numerous hurdles. First, the genome consists of complex epistatic networks of genes. Second, each gene can have pleiotropic effects on multiple traits. Third, the causal path between a gene and its downstream phenotype is far from direct, including effects on the transcriptome, proteome, and metabolome, and influenced by environmental effects. This figure suggests that the metabolome can provide a valuable intermediate stage to complete the G-P map, identifying genetic and environmental factors that influence metabolomic profiles, and then correlating metabolomic profiles with phenotypes of interest.

Here, we suggest that many of the challenges that we face in our attempts to define the pathways that account for age-related declines in function, and for genetic variation in these declines, can be resolved through the use of high-resolution metabolomics (Mishur & Rea, 2012). If we can decompose the physiological processes that influence morbidity and mortality to their constituent components (i.e., the metabolome), we will be an important step closer to bridging the gap between genotype and phenotype (Fig. 1). The metabolome is effectively a functional intermediate between genotype and phenotype.

Previous work illustrates how the metabolome can serve as a strong bridge between genotype and phenotype. While allelic variation typically explains only a small fraction of the variation in complex phenotypes, GWA studies of the metabolome have found genetic variants capable of explaining up to 60% of the variance in the concentration of individual metabolites (Suhre *et al*., 2011). The metabolome also appears to be a sensitive indicator of age-related physiological changes both in invertebrates and vertebrates (e.g., Sarup *et al*., 2012; Yu *et al*., 2012). Moreover, different mutants that extend longevity share common metabolomic signatures (e.g., *Caenorhabditis elegans*: Fuchs *et al*., 2010; *Mus musculus*: Wijeyesekera *et al*., 2012).

While we have learned much from these initial studies, most have been limited by the use of relatively low-sensitivity metabolomics technology and by limited genetic information. Studies of age and the metabolome have hitherto been carried out using standard metabolomic methods, which typically measure concentrations of several hundred metabolites, at most. This represents one percent or less of all the circulating metabolites found within an individual animal (Jones *et al*., 2012). This would be the equivalent of measuring just 300 genes in a ‘genome-wide’ screen in humans. Moreover, most of these studies have not been able to distinguish metabolomic variation that is due to genetic differences among individuals from variation due to nongenetic physiological differences.

Here, we demonstrate the power of new, state-of-the-art high-resolution metabolomics (Jones *et al*., 2012) to characterize the aging metabolome. Hardware and software advances in high-resolution metabolomics now support extremely sensitive measurement of tens of thousands of metabolites (Jones *et al*., 2012; Uppal *et al*., 2013), and these high-resolution studies can be used to determine the effects of age on the entire metabolome (De Guzman *et al*., 2012). We use high-resolution metabolomics to ask three specific questions. First, is the metabolome a sensitive indicator of physiological state in the aging fly? Second, is the high-sensitivity metabolome a useful biomarker of age, can it predict the age of an individual, and moreover, can it explain sex- and genotype-specific variation in age. And third, can metabolomic studies reveal novel pathways associated with natural variation in aging?

## Results

We analyzed metabolomics data from 15 inbred lines from the *Drosophila* Genome Reference Panel (DGRP, Mackay *et al*., 2012) (Table S1, Supporting information). After quality control (see Experimental procedures), our final dataset consisted of 293 biological samples, each of which was run twice through each of two columns. Each sample provided measures for 3091 features from an anion exchange (AE) column, and 3714 features by reverse phase (C18) liquid chromatography, for a total of 6805 features (note that some overlap occurs between columns) (see Experimental procedures below, and Soltow *et al*., 2011; for a description of the differences between these two columns). Of the features detected, we were able to obtain putative molecular matches for 877 metabolites in the AE column and 717 metabolites in the C18 column based on mass-charge (*m*/*z*) ratio using the metabolite prediction program *mummichog* (Li *et al*., 2013). In our individual metabolite analysis, we define statistically significant effects on *m*/*z* concentrations using a conservative false discovery rate α = 0.01 (Benjamini & Hochberg, 1995). Seven of the fifteen lines used here were putatively positive for *Wolbachia* infection (Table S1). Including *Wolbachia* status in our statistical models had no appreciable effect on our conclusions and so is not discussed further.

We divide the presentation of results into three sections. First, we examine the broad effects of age, sex, and genotype (including measures of heritability) on individual metabolites. Second, we identify metabolites that show significant interaction effects, looking in particular at age × sex and age × genotype interactions. Third, we describe specific types of metabolites that are enriched among all metabolites significantly associated with these main effects and their interactions.

### Main effects

**Figure 2:**
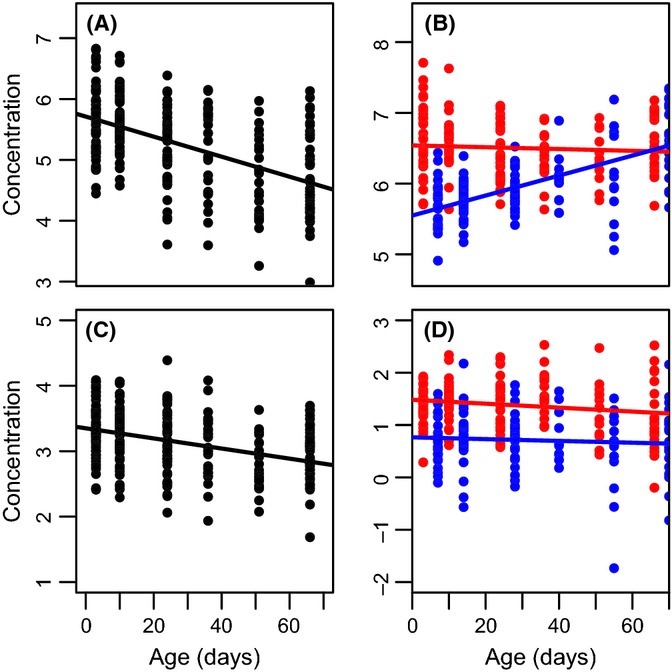
Specific metabolites associated with age (A, C), sex (D), and their interaction (B) (*P* < 4 × 10^−11^ in all cases). Blue dots indicate males, and red dots indicate females; the value on the age-axis for (B) and (D) is shifted between the sexes for illustrative purposes only. Confirmed metabolite identities include (A) oleoylcarnitine; (B) 1-oleoylglycerophosphoethanolamine; (C) stearoylcarnitine; and (D) glutamine.

The metabolome appears to be highly sensitive to physiological state, showing dramatic variation in response to sex, age, and genotype (Table 1). The proportion of metabolites significantly associated with the traits we measured varied from a low of 1% of C18 metabolites increasing with age to a high of almost 14% of AE metabolites that vary among genotypes. In each case, effects of one variable were measured after controlling statistically for the effects of the other two variables. For example, our test for the effect of genotype held the effects of sex and age constant, effectively testing if there are significant differences in the intercept of *m/z* intensity vs. age among genotypes. Figure 2 shows two metabolites with confirmed identities with significant age effects and two with significant sex effects. Note that sex effects are likely underestimated, because we only included metabolites present in at least 95% of male samples and 95% of female samples.

**Table 1:** Number and percentage of metabolites associated with age, sex, genotype, and their interactions for anion exchange (AE) and C18 columns

Parameter	# of AE metabolites	% of AE total	# of C18 metabolites	% of C18 total
Increase with age	76	2.5	36	1.0
Decrease with age	96	3.1	167	4.5
Increase in males	267	8.6	133	3.6
Increase in females	381	12.3	431	11.6
Genotype	423	13.7	292	7.9
Age × sex	71	2.3	167	4.5
Age × genotype	137	4.4	542	14.6
Sex × genotype	26	0.8	15	0.4

As a separate measure of genetic effects, we used the intraclass correlation (*t*) among the fifteen inbred lines as a measure of heritability (the percentage of total variation due to genetic effects). We identified almost 300 metabolites with *t* ≥ 5% (134 C18 metabolites, 149 AE metabolites), with maximum value of *t* = 28.8% among AE metabolites (*m/z* = 693.2182) and *t* = 32.3% among C18 metabolites (*m/z* = 179.0844). The full distribution of heritabilities is shown in Fig. S1 (Supporting information).

**Figure 3:**
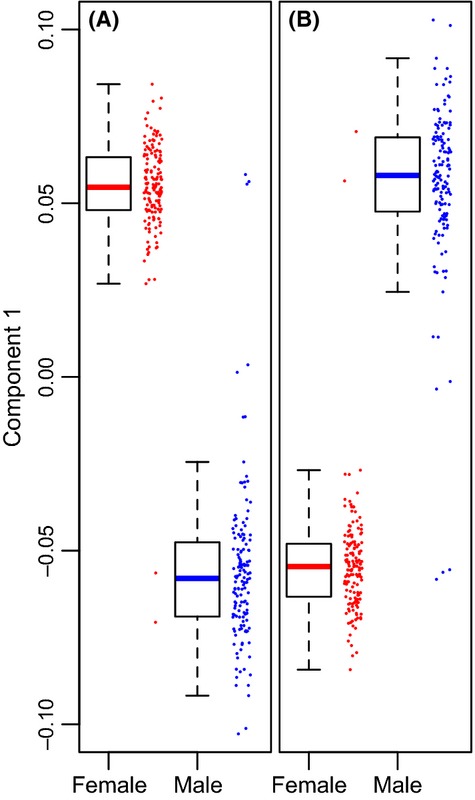
First component value from partial least squares discriminant analysis showing strong separation of males and females. (A) anion exchange column [minimum classification error rate (CER) based on ten-fold validation = 0.017, using four metabolites]. After permuting the class variable (sex), CER = 0.385 ± 0.008 (mean ± standard error, *n* = 10 permutations). (B) C18 column (minimum CER = 0.021 based on 25 metabolites. After permutation, CER = 0.371 ± 0.015 [mean ± SE, *n* = 10 permutations]).

**Figure 4:**
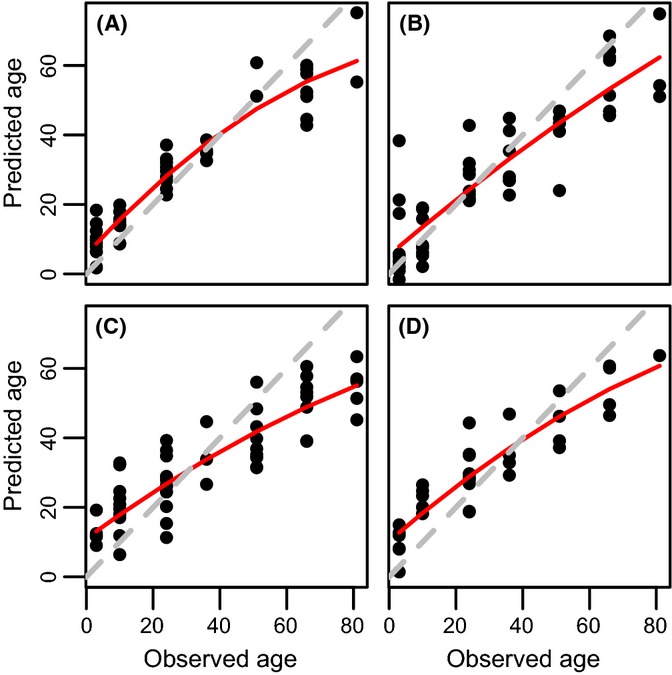
Predicted vs. observed age, for C18 males (A), anion exchange (AE) males (B), C18 females (C), and AE females (D). In each case, two-thirds of all samples were chosen at random to construct a model using partial least squares regression. This model was then used to predict values for the remaining one-third of the samples. Predictions were repeated twenty times in each case, and the figures shown here represent cases close to the mean *R*^2^ value. The red line represents a second-order polynomial fit to the data, and the dashed gray line is the isometric line. These results are consistent with *R*^2^ values obtained from ten-fold cross-validation scores based on sparse PLS on the full dataset (*R*^2^ values: AE males: 0.92; AE females 0.83; C18 males: 0.88; C18 females: 0.78). In all four cases, *R*^2^ values for permuted datasets were < 0.05.

Supervised multivariate analysis revealed that the metabolome is strongly predictive of sex and age. Partial least squares discriminant analysis differentiated almost completely between the metabolome of males and females (Fig. 3) and among flies of different ages (Fig. S2, Supporting information). Similarly, using partial least squares regression, we found the metabolome to be an accurate predictor of age. For both males and females, a model based on a random sample of two-thirds of all individuals was able to explain between 78% and 92% of the variance in age of the remaining one-third of samples (Fig. 4).

### Interaction effects

In addition to the substantial proportion of metabolites that showed significant age-specific changes in intensity or that differed significantly between sexes or genotypes, we also found numerous metabolites associated with interaction effects [i.e., metabolites for which the slope of change with age differs significantly between the sexes or among genotypes (Table 1)]. One example of a known metabolite with significant effects on age, sex, and their interaction is shown in Fig. 2B.

Taken together, we found a total of 995/3091 metabolites (32.2%) in the AE column and 995/3714 (26.8%) in the C18 column whose concentration was effected by age, sex, genotype, or some interaction thereof.

### Metabolite enrichment

Using the program *mummichog* (Li *et al*., 2013), we asked whether subsets of metabolites associated with a particular trait or trait combination were enriched for specific classes of metabolites. Our analysis revealed a large number of metabolic pathways whose constituent metabolites were overrepresented among all metabolites associated with sex, age, and their interactions. The complete list is shown in Table S2 (Supporting information).

**Figure 5:**
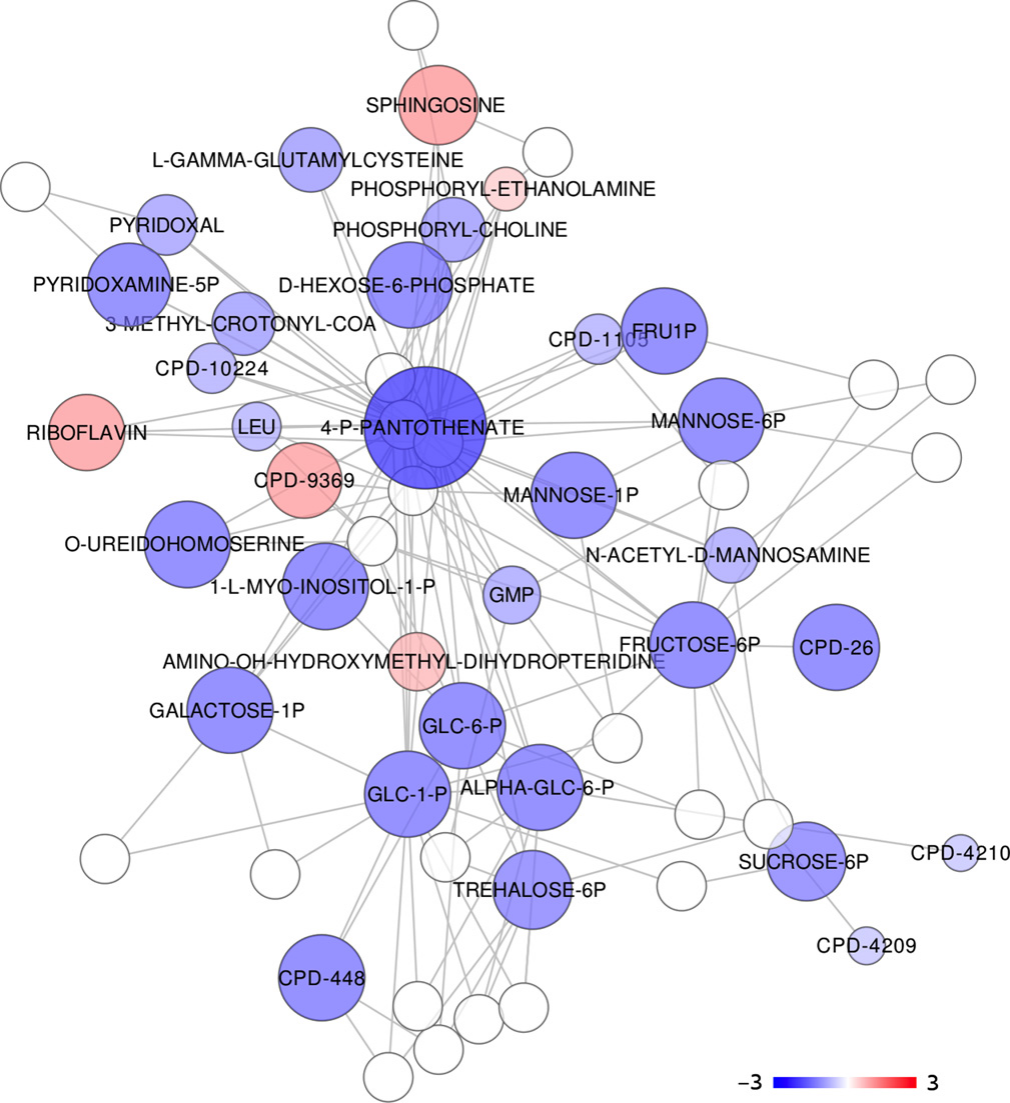
The network shown here represents output from *mummichog* analysis with color hue determined by the sign and size and color intensity determined by the magnitude of the regression coefficient in the age model (blue is negative, red is positive). The metabolites are putatively annotated based on *m/z* ratio. This particular module is enriched for metabolites associated with glycolysis, for metabolites that feed the glycolytic pathway, and for metabolites associated with glycophospholipid metabolism (*P* = 0.04, see text for details).

Among metabolites that change with age, we identified four groups that are notable for being strongly enriched for certain pathways and being of specific interest from an aging perspective. The first group, illustrated in Fig. 5, includes metabolites associated with sugar and glycerophospholipid metabolism. These two pathways are connected via interactions with 4′-phosphopanthothenate. Metabolites associated with glycolysis, including G6P, F6P, and ‘feeder’ molecules F1P, G1P, galactose 1-phosphate, trehalose 6-phosphate, and mannose 6-phosphate, show a clear decline with age. Metabolites associated with glycerophospholipid metabolism show both increases and declines with age and include sphingosine, phosphoryl ethanolamine, 1-l-myo-inositol-1-phosphate, and phosphoryl choline.

The second group of molecules is the carnitines, illustrated in Fig. S3 (Supporting information). These molecules, which make up the carnitine shuttle, are a key component of fatty acid metabolism and show an almost uniform, highly significant decline with age.

The third and fourth groups, described in detail in Table 2, include amino acids (AA) and neurotransmitters, including molecules involved in monoamine metabolism as well as the inhibitory neurotransmitter γ-aminobutyric acid (GABA) and its precursor arginine.

**Table 2:** List of specific amino acids (AA) and neurotransmitters associated with different age parameters

Metabolite	Effect
AAs	
Tryptophan	Age ↑, Age ↓, A × G
Isoleucine/leucine	Age ↑, Age ↓, A × G
Arginine	Age ↑
Phenylalanine	Age ↑
Methionine	A × S
Proline	A × S, A × G
Threonine	A × S, A × G
Aspartate	A × G
Glutamine	A × G
Neurotransmitters	
*N*-acetyl-serotonin	Age ↓
4-alpha-Hydroxytetrahydrobiopterin	Age ↑, A × S
5-hydroxy-l-tryptophan	Age ↑, A × S
Tetrahydrobiopterin	A × G
l-Dopa	Age ↓, A × S
Dopamine	Age ↑
γ-Aminobutyric acid	Age ↑, Age ↓

Age ↑ and Age ↓ refer to metabolites that either increase or decrease with age, respectively. A × S and A × G refer to metabolites with significant interaction effects between age and sex or age and genotype, respectively. Effects are all significant after correction for multiple comparisons with a false discovery rate of 0.01 (see text). Note that isoleucine and leucine co-elute under our conditions and have identical mass-charge ratios so cannot be distinguished.

Among the 150 most heritable metabolites, we observed enrichment for two pathways related to tryptophan metabolism–tryptophan degradation (AE: *n* = 4 observed/8 total, *P*_adj_ = 0.0034; C18: *n* = 2 observed/7 total, *P*_adj_ = 0.023) and serotonin and melatonin biosynthesis (AE: *n* = 4/6, *P*_adj_ = 0.0028; C18: *n* = 2/7, *P*_adj_ = 0.023).

## Discussion

Beginning with Pletcher *et al*.’s (2002) groundbreaking study on the transcriptome of aging fruit flies, systems biological studies of aging in the fruit fly have focused primarily on the transcriptome. Here, we turned our attention to the metabolome, in an attempt to determine the degree to which the metabolome (i) is associated with physiological state; (ii) can predict age, sex, and genotype-specific variation in aging; and (iii) might reveal novel metabolic pathways associated with natural genetic variation in aging.

While previous studies have looked at the effects of age, sex, or genotype on metabolomic variation, this study is unique not only in its scope (i.e., the very large number of metabolites analyzed), but also in its design. By including age, sex, and genotype, we were able to measure the independent effects of all three of these fundamental biological parameters simultaneously, as well as of their interactions, and to identify specific metabolic pathways associated with changes due to age, sex, and genotype. This study also takes advantage in particular of new advances in high-resolution metabolomics (Jones *et al*., 2012). With the availability of this new system, we can now identify tens of thousands of features from samples as small as a few flies (2–3 mg of tissue, in this case).

We found that the metabolome is, indeed, highly sensitive to physiological state as influenced by sex, by age, and by underlying genotype, with as much as one-third of the entire metabolome responding significantly to these factors or their interactions. Moreover, the metabolome proved to be strongly predictive of sex and age. These findings hold out the hope that metabolomic profiles might be a powerful biomarker of age. Further studies are needed to determine whether samples that are younger or older than predicted by their metabolomic profile would be relatively longer- or shorter-lived, respectively.

While this study set out to identify metabolites affected by age, the most dramatic effects were found with respect to sex. In fact, we have likely underestimated the proportion of metabolites that differ between the sexes, as we excluded all metabolites that were absent in more than 5% of samples from either sex. Thus, metabolites that were found consistently in one sex, but less so or not at all in the other sex, would have been excluded from our analysis. Our finding of sex differences in multiple classes of metabolites is consistent with earlier studies on flies showing sex differences in lipid profiles (Parisi *et al*., 2011; Scheitz *et al*., 2013).

Given these extensive differences, and the ease with which we are able to carry out genetic manipulations in *Drosophila*, we should be able to use the fly as a model system to identify the genetic basis of sex-specific differences in the metabolome.

We found between 7% (C18) and 13% (AE) of all metabolites differed among genotypes. This finding holds out the promise of addressing two critical issues. First, given the strong genetic signature of variation in metabolites, we should be able to identify the genetic basis of individual variation in metabolites in *Drosophila*, as others have carried out in humans (e.g., Kettunen *et al*., 2012; Suhre & Gieger, 2012). Second, and of central interest to aging research, we should be able to determine the extent to which genotype-specific metabolite profiles are correlated with lifespan (and, of course, other traits of interest). These results also fit well within the context of earlier work showing the effects both of life-extending mutants (e.g., Fuchs *et al*., 2010; Wijeyesekera *et al*., 2012) and of selection on lifespan (Sarup *et al*., 2012) on metabolite profiles. Whether these treatments can actually reverse the effects of age on the metabolome requires more detailed analysis.

Perhaps the most unexpected finding was the considerable number of metabolites that showed significant age × genotype interactions (Table 1). This finding was facilitated by the fact that we were able to measure metabolites at seven different ages for 15 different genotypes. Scaled up to a larger sample of genotypes, we are confident that future studies should enable us to identify single genes associated not only with baseline metabolite levels (e.g., Suhre *et al*., 2011; Kettunen *et al*., 2012), but also with the degree to which metabolite concentrations change with age and the impact of these changes on other age-related traits.

The strong effect of a sex × age interaction on metabolomic profiles also holds out great promise for our ability to better understand why sexes differ in longevity and in patterns of age-associated disease. Previous studies have looked at the degree to which age and sex affect metabolite concentration (Slupsky *et al*., 2007; Psihogios *et al*., 2008), although these studies have not looked specifically at *interactions* between sex and age. Going a step further, we might ask whether we can identify genes that affect sex differences in age-specific trajectories of specific metabolites. While such a ‘third-order’ analysis is complex, its feasibility is hinted at by the existence of numerous metabolites with statistically significant three-way interaction between sex, age, and genotype (C18: *n* = 56; AE: *n* = 34).

Our findings suggest not only that the metabolome might be a useful biomarker of physiological state, but also that it might reveal novel pathways associated with age. The age-related decline in carnitines was perhaps the most consistent pattern that we observed here. Carnitines are critical for the transfer of fatty acids into the mitochondrian, where they undergo β-oxidation, generating acetyl-CoA, which then enters the TCA cycle. In their study of aging in mice, Houtkooper *et al*. (2011) found a similar age-related decline not only in acylcarnitines, but also in genes associated with fatty acid metabolism. Similar declines have also been seen in humans (e.g., Gomez *et al*., 2012), and numerous studies point to the ameliorative effects of supplemental carnitines on senescence (e.g., Noland *et al*., 2009). Our findings are consistent with another recent study in *Drosophila* showing age-related changes in fatty acid profiles (Moghadam *et al*. 2013).

Amino acid balance is thought to explain the dramatic response of lifespan to dietary restriction (Grandison *et al*., 2009), and AAs are important activators of the aging-related target of rapamycin (TOR) pathway. We found AA enrichment among metabolites that increased with age, that decreased with age, that were higher in males, and that interacted between age and sex or age and genotype. These last two groups suggest that AA levels might be useful in predicting differences in patterns of aging between males and females, or among genotypes. In support of this potential, AAs were among the metabolites with the highest heritability, a finding consistent with previous studies on the heritability of the metabolome (Suhre *et al*., 2011; Kettunen *et al*., 2012; Rhee *et al*., 2013). Interestingly, the AAs that we identified as having the highest heritability were similar to three of four AAs found to be highly heritable in humans, including isoleucine, proline, and glutamine (Suhre *et al*., 2011).

Among metabolites that declined with age, we also observed enrichment of those associated with glycolysis and glycophospholipid metabolism. These two groups were linked through pantothenic acid (Vitamin B), which is required for the conversion of pyruvate (from glycolysis) to acetyl-CoA, part of phospholipid biochemistry. Our data suggest that at least in flies, glycolysis declines with age. We saw that overall, membrane phospholipids declined with age, although some metabolites, such as phosphatidylethanolamine and phosphatidylcholine, moved in opposite directions (Fig. 5), an observation consistent with previous studies (Kostal *et al*., 2011). Studies in flies have shown strong effects of thermal stress on membrane phospholipids (e.g., Overgaard *et al*., 2008). Future studies might benefit from a focus on the degree to which the well-established effect of temperature on lifespan is associated with changes in phospholipid biochemistry.

In this study, biogenic amines were associated with age and sex, and tryptophan metabolism in particular showed high levels of heritability. Previous studies in flies have suggested that both dopamine and serotonin signaling might be important regulators of aging (De Luca *et al*., 2003; Vermeulen *et al*., 2006), either directly or indirectly through their interaction with insulin signaling pathways (Toivonen *et al*., 2009).

Interestingly, we also saw enrichment for the tryptophan degradation pathway, to kynurenine, among metabolites with the highest heritability. Recent work has implicated this pathway with aging in both worms (Coburn *et al*., 2013) and flies (Oxenkrug *et al*., 2011).

Given the enrichment of these pathways among the most heritable metabolites, our results offer hope that these pathways might help to explain variation in important fitness traits, including survival, in natural populations. This might be especially true of sex-specific differences in aging. Biogenic amines were found to be common not only among metabolites that differed between males and females, but also among metabolites whose age-specific trajectories were sex-specific (Table S1).

There are, of course, some limitations to this work. First and foremost, we do not yet have a curated *Drosophila* metabolome. While we are able to obtain putative matches for approximately one-quarter of all metabolites, these matches often carry considerable uncertainty. In some cases, a single mass-charge ratio might match 10 or more putative known molecules. Once the fly metabolome is curated, we will be able to more accurately describe the dynamics of fly metabolic pathways. Moreover, in limiting our assay to metabolites with *m/z*-ratios of < 900, we have eliminated many components of lipid metabolism, which is of interest to aging studies (Barzilai *et al*., 2001).

Second, our study relied on whole flies. We know that metabolomic profiles differ among tissues in the fly (Chintapalli *et al*., 2013). Moreover, our whole-body analysis includes the gut and so includes potentially confounding metabolites from the large bacterial flora found in the fly gut (Corby-Harris *et al*., 2007).

Third, while this is the largest-scale analysis of age-related change in the metabolome to date, the data represent just one experiment (albeit a large-scale one). Changes that appear to be related to age might, in fact, be due to secular environmental trends that occurred over the 12-week course of the experiment (an often unstated concern of cohort aging studies).

Our work has established the power and potential of the fly metabolome as a model to both explain and predict variation in aging. In light of our findings, there are several avenues for future research of immediate interest. To begin with, we have established that there is substantial genetic variation in the metabolome and that a large number of specific metabolites are not only affected by age, but also show age effects that vary by genotype. Thus, we are confident that we can use metabolomic variation to tie together genotype with phenotype, identifying genes that affect metabolites and metabolites that affect and/or reflect variation in lifespan. In this way, large-scale metabolomic studies hold out much promise in helping us to complete the genotype–phenotype map for aging. Furthermore, here we have focused on measures of individual metabolites. Recent work suggests that as individuals age, we see changes not only in the levels of specific molecules, but also in the way that these molecules interact with one another within intracellular networks (Soltow *et al*., 2010). The metabolomic profiles we have described here should allow us to examine age-specific changes in network structure, and in so doing, to generate novel hypotheses regarding pathways that are robust or frail in the face of the aging process. Finally, and as we mentioned earlier, we must now make it an urgent priority to curate the metabolomes of all model organisms that are used in aging research. A recent curation of the yeast metabolome has been made available (Jewison *et al*., 2012). To this, we now need to add the worm and the fly.

## Experimental procedures

### Fly stocks and culturing conditions

All analyses were carried out using a set of 15 inbred fruit fly genotypes randomly chosen from the DGRP (Mackay *et al*., 2012) (Table S1). The DGRP consists of 192 fully inbred and fully sequenced strains of *Drosophila melanogaster* and is freely available from the Bloomington *Drosophila* Stock Center. Flies were maintained in incubators at 24 °C on a 12/12 light–dark cycle at ~50% humidity. For all procedures, flies were maintained on standard yeast-molasses-agar-cornmeal medium.

### Collection of known-age flies

Prior to the onset of the study, fly cultures were expanded to include four bottles per genotype, at which point 150 virgin males and females were collected over a 72-h period under light CO_2_ anesthesia. For each sex and each of the 15 genotypes, we placed an average of 27 individuals in each of 5 40-mL glass vials, for a total of 4032 flies distributed among 150 vials. Flies were transferred to new vials very 2 days without anesthesia, at which time the number of dead flies in each vial was recorded.

At seven time points (days 3, 10, 24, 36, 51, 66, and 81), we collected two samples of three flies from each unique genotype-sex cohort without anesthesia, placed each sample in a 1.5-mL Eppendorf tube, instantly froze the samples in liquid nitrogen, and then placed these samples in a −80 °C freezer until the end of the experiment. Not all genotypes survived to age 81 day, and in some cases at later ages, only one sample of three flies per genotype and sex was collected.

### Metabolomic analysis

Each frozen fly sample was homogenized using a Pellet Pestle® Motor (Kimble Chase, Vineland, NJ, USA) in 150 μL acetonitrile in water (2:1 v/v) containing an isotopic standard mix (Soltow *et al*., 2011) and refrozen. Immediately before analysis, the samples were thawed, vortexed, and centrifuged at 12 300 *g* for 10 min at 4 °C. Extracts (100 μL) were randomized, placed in a refrigerated autosampler, and 10 μL volumes were analyzed in duplicate with dual chromatography-mass spectrometry (DC-MS) platforms (Soltow *et al*., 2011), one using an AE column (PRPX-110S, 2.1 mm × 10 cm; Hamilton Company, Reno, NV, USA) and the other using a C18 column (Targa, 2.1 mm × 10 cm; Higgins Analytical, Mtn View, CA, USA). C18 and AE chromatography separate molecules based upon different chemical properties. C18 is also termed ‘reverse phase’ chromatography because the 18-carbon units are hydrophobic, retaining and separating chemicals with partial hydrophobic character. Anion exchange, on the other hand, has positive charges on the column, which retain and separate negatively charged molecules. The conditions used result in about 30% overlap between columns in chemicals detected.

Samples were fractionated with a formate or acetonitrile gradient, respectively, ionized with electrospray ionization in the positive mode, and detected with an LTQ Orbitrap Velos mass spectrometer (Thermo Fisher Scientific, San Jose, CA, USA) with *m/z* from 85 to 2000 and 30 000 resolution. Data were extracted using apLCMS (Yu *et al*., 2009) as *m/z* features, where an *m/z* feature is defined by *m/z* (mass/charge), retention time, and ion intensity (integrated ion intensity for the peak). As part of quality control, we also generated a ‘fly standard’, consisting of a large volume of identical sample taken from 350 flies, which was run alongside samples daily to evaluate reproducibility. While some overlap in metabolites between the two columns is expected, data from both columns cannot efficiently be combined for analysis due to very different column chemistries which can lead to the same metabolite showing different ion intensities and retention times. Therefore, we analyzed data from both columns separately.

### Data analysis

#### Quality control

Many metabolites show significant stochastic variation even within samples and thus are likely to be uninformative. To minimize the impact of ‘noisy’ metabolites, prior to data analysis, we carried out a set of quality control procedures to limit our analysis to the most informative metabolites. First, we only included metabolites with a signal-to-noise ratio (SNR_*i*_ = mean/sample standard deviation) ≥ 15. Second, we log-transformed the data, which led to a normal distribution of concentrations across all metabolites. Third, we removed any metabolites that were missing from more than 5% of either all male samples or all female samples. Fourth, we used the LSimpute imputation procedure (Bo *et al*., 2004) to estimate the values of missing samples. Fifth, we limited our analysis to metabolites with a mass-charge ratio of < 900, as data collection parameters were optimized for *m*/*z* ≤ 900. Finally, once all these procedures were complete, we normalized the data such that each sample had a mean value of 0.

#### Metabolite-specific analysis

All statistical analyses were carried out using the statistics package R (R Core Team, 2013).

We used a general linear model to test for the effects of age (*A*), sex (*S*), and genotype (*G*) on metabolite intensity (*Y*):

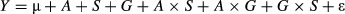
(1)treating all predictors as fixed effects, where μ is the grand mean and ε is the residual error. We treated age as an ordered factor (effectively a categorical variable, with the proviso that we know that age 3< age 10, age 10< age 24, and so forth). Due to small sample sizes and not all genotypes being present, age 81 flies were removed, leaving 274 samples for metabolite-specific analyses.

By including all parameters in the model, we are asking, for example, whether sex has a significant effect on metabolite intensity after controlling for the effects of age and genotype. To determine significance for each factor in the model shown in Eqn 1, we set the false discovery rate (Benjamini & Hochberg, 1995) at 1% using all *P*-values associated with that specific factor. To obtain *P*-values for the effects of genotype and of its interactions with age or sex, we carried out a likelihood ratio test using the lrtest function in the *epicalc* package in r.

We followed Hoffmann & Parsons (1988) to obtain approximate estimates of narrow-sense heritability (*h*^2^) from the intraclass correlation (*t*) between lines. Here, 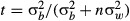, where 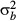 and 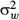 are the between-line and within-line variances, respectively, and *n* = 3 is the number of individuals within each sample. Within- and between-genotype variances were determined using the lme function in the package nlme in r, treating age and sex as fixed effects and genotype (line) as a random effect. While Hoffmann & Parsons (1988) and subsequent authors suggest various equations to convert *t* to *h*^2^, here we present only the intraclass correlation.

#### Metabolome-wide analysis

To determine the degree to which metabolomic profiles could be used to predict sex or age, we used the sparse partial least squares discriminant analysis function (splsda) as implemented in the r package *mixOmics* (for sex and genotype) and partial least squares regression as implemented by the mvr function in the *pls* package in r (for age). We set the number of components as (*k*−1), where *k* is the number of classes (2 for sex, 7 for age, 15 for genotype). For splsda, we chose the number of metabolites to include in each analysis based on that number which minimized the classification error rate (CER), using ten-fold cross-validation. Supervised classification schemes with relatively low numbers of samples and high numbers of variables can lead to overfitting (Westerhuis *et al*., 2008). Accordingly, we compared observed CER with mean CER (±1 SE) obtained from ten permutation tests in which the classification group (sex or age) was sampled randomly without replacement. This comparison tells us whether the observed classification is any better than one would expect by chance.

We used partial least squares to predict sample age using training and testing sets. In this case, training sets consisted of 2/3 of all samples, and the model derived from this set was used to predict the classification for the remaining 1/3 of the samples. We obtained *R*^2^ values using ten-fold cross-validation and compared these values with *R*^2^ values where the class variable was permuted.

#### Metabolite annotation and tests of metabolite enrichment

In this study, we adopted a novel approach to perform putative metabolite annotation and enrichment analysis in one step (Li *et al*., 2013). Theoretically, most metabolites from mass spectrometry can match multiple metabolite compounds. The program that we have used here, *mummichog*, selects the most probable metabolites from these multiple candidates, based on the enrichment of metabolic networks and pathways, because a biological process is expected to favor a more connected network over random distributions. This approach has been validated on multiple experimental datasets (Li *et al*., 2013). The fly metabolic network model from the MetaCyc database (Caspi *et al*., 2012) and the reference model in *mummichog* (Li *et al*., 2013) were used to test the enrichment of pathways and networks. The null distribution in pathway analysis is based on the permutation of metabolite selections, which takes into full consideration the aforementioned multiple-matching problem.

We first determined subsets of metabolites to look for pathway enrichment. We ran a model for both columns separately of only age, sex, and their interaction, and took the 250 most positively and 250 most negatively associated metabolites based on their *P*-value for each parameter (age, sex, and the interaction between the two). This gave us 12 different subset lists (six from each column) that were then run through the program *mummichog* for pathway enrichment analysis.

For age and genotype interactions, we ran a likelihood ratio test as described above to determine those metabolites with a significant age–genotype interaction. We chose 250 metabolites with the smallest *P*-value for both the C18 and AE columns for enrichment analysis.

## Supporting Information

**Fig. S1** Histogram of intraclass correlations among all metabolites for AE (top) and C18 (bottom) columns.

**Fig. S2** Sparse partial least squares discriminant analysis is able to distinguish samples of different age, where age is treated as a factor rather than a continuous variable as in Fig. 4, with relatively high accuracy.

**Fig. S3** Carnitine shuttle pathway from *mummichog* analysis of metabolites, with color and intensity determined by the sign and magnitude of the regression coefficient in the age model (blue is negative, red is positive).

**Table S1** Bloomington Stock Center numbers, DGRP numbers, and wolbachia status for those inbred lines analyzed in this study.

**Table S2** *Mummichog* enrichment analysis for sets of analytes significantly associated with specific factors.
