# Supplementary figures and images for: Effects of age, sex, and genotype on high-sensitivity metabolomic profiles in the fruit fly, Drosophila melanogaster
Source: Aging Cell. 2014 Mar 18;13(4):596–604. doi: 10.1111/acel.12215 (PMC4116462; doi:10.1111/acel.12215)

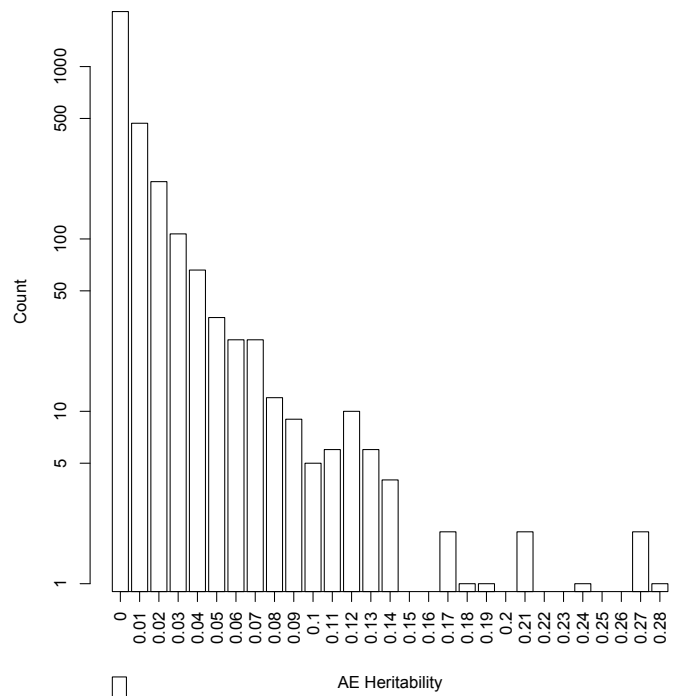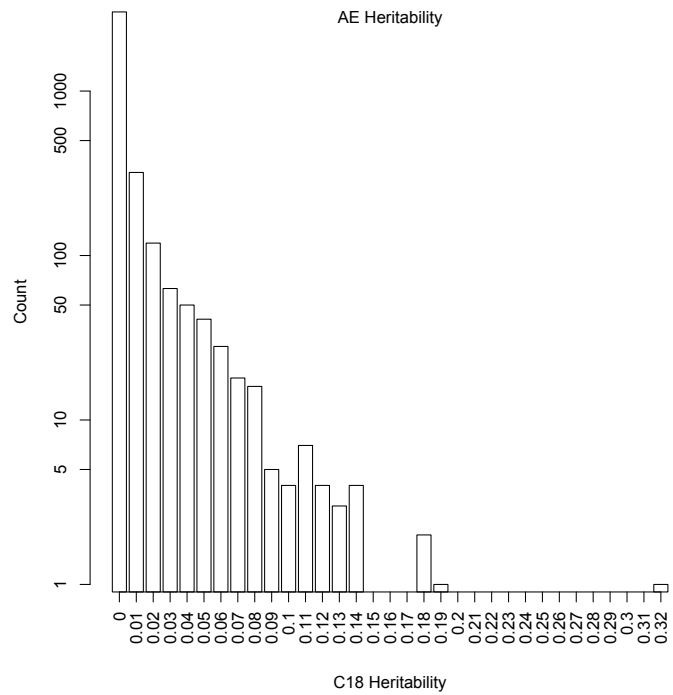

Supplement: Supplementary file 1 — Fig. S1 Histogram of intraclass correlations among all metabolites for AE (top) and C18 (bottom) columns. [file acel0013-0596-sd1.pdf]

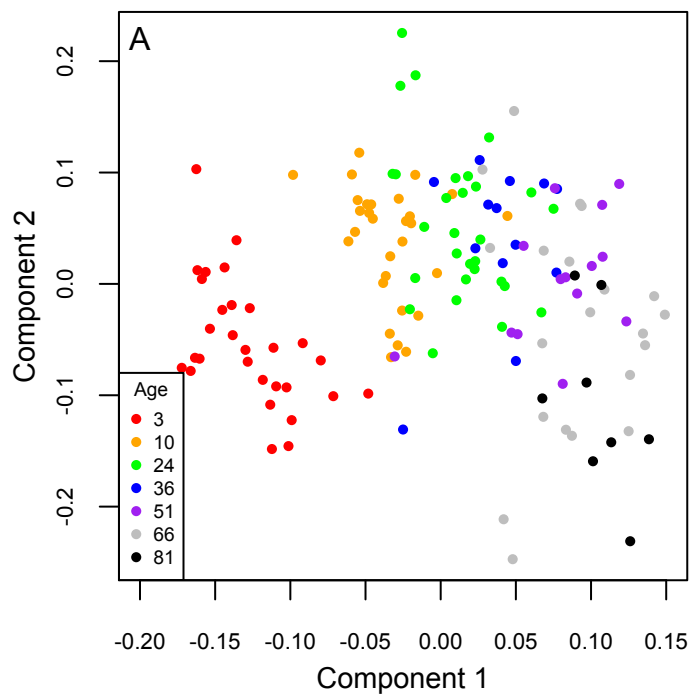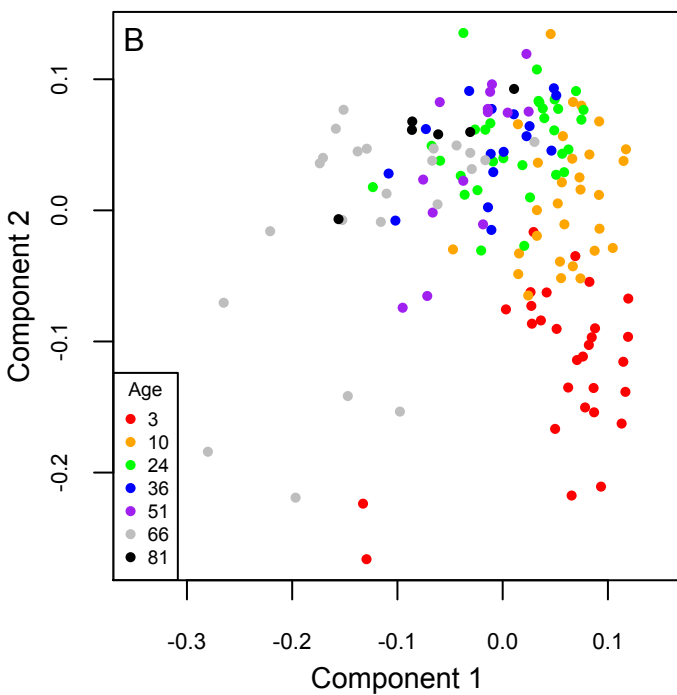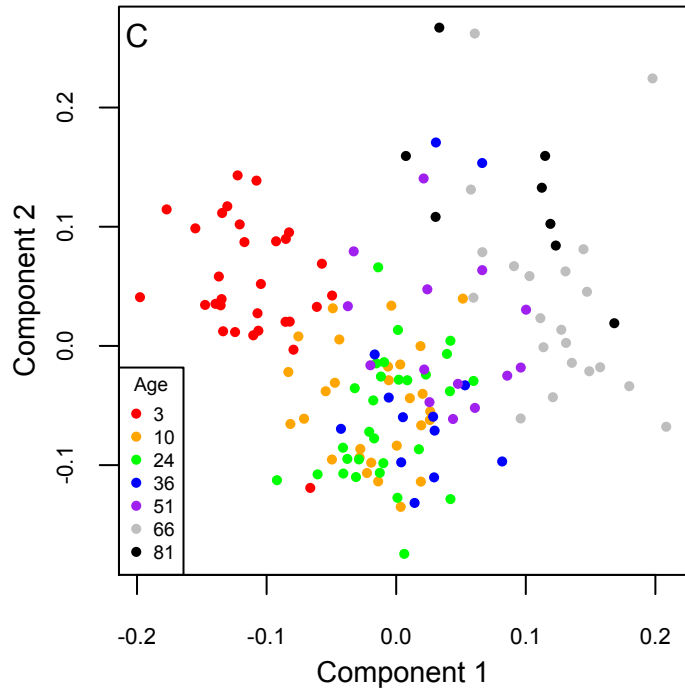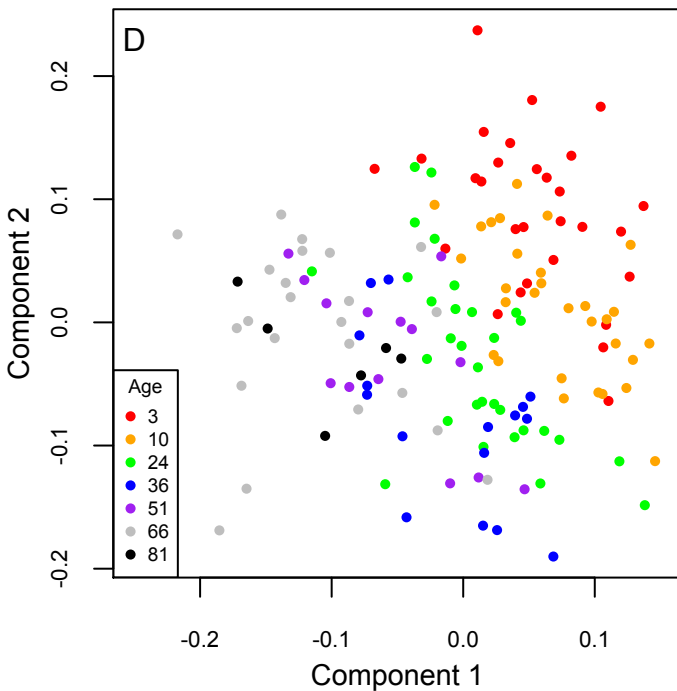

Supplement: Supplementary file 2 — Fig. S2 Sparse partial least squares discriminant analysis is able to distinguish samples of different age, where age is treated as a factor rather than a continuous variable as in Fig. 4, with relatively high accuracy. [file acel0013-0596-sd2.pdf]

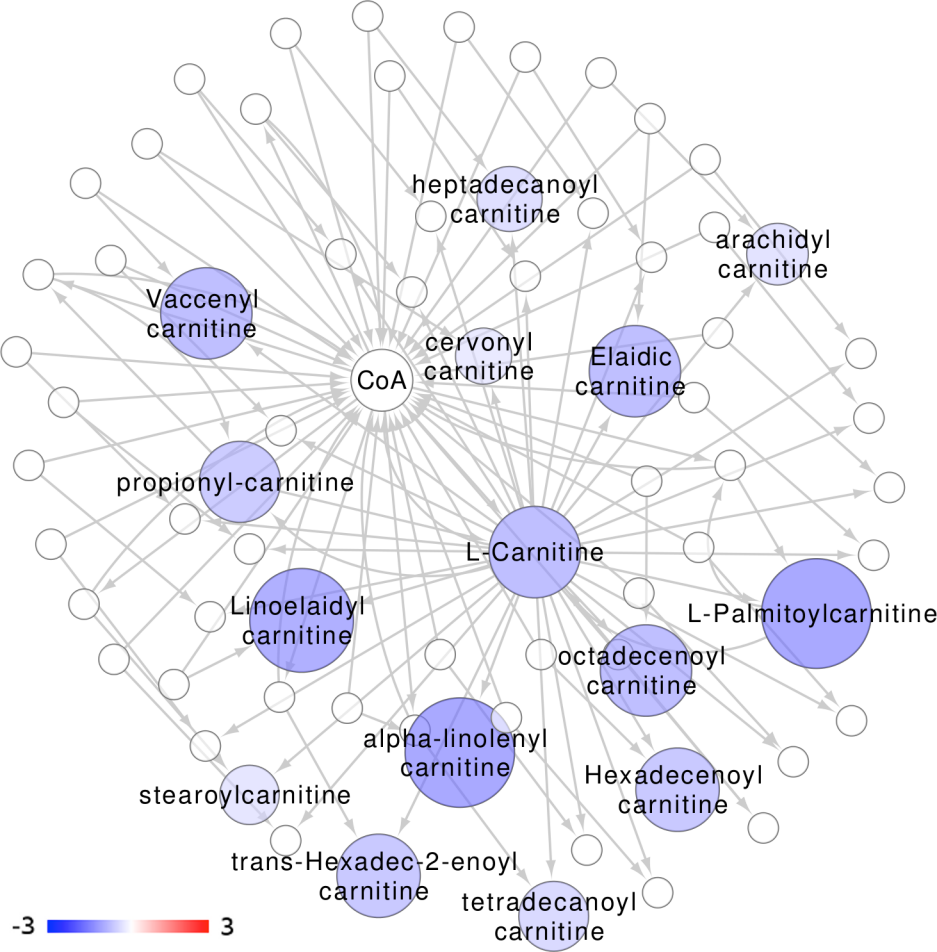

Supplement: Supplementary file 3 — Fig. S3 Carnitine shuttle pathway from mummichog analysis of metabolites, with color and intensity determined by the sign and magnitude of the regression coefficient in the age model (blue is negative, red is positive). [file acel0013-0596-sd3.pdf]
